# Transgender‐based disparities in suicidality: A population‐based study of key predictions from four theoretical models

**DOI:** 10.1111/sltb.12830

**Published:** 2022-01-23

**Authors:** Richard Bränström, Isabella Stormbom, Morgan Bergendal, John E. Pachankis

**Affiliations:** ^1^ Department of Clinical Neuroscience Karolinska Institutet Stockholm Sweden; ^2^ Department of Social and Behavioral Sciences Yale School of Public Health New Haven Connecticut USA

**Keywords:** LGBTQ, minority stress, suicidality, transgender

## Abstract

**Introduction:**

Numerous studies have reported a high prevalence of suicidality among transgender individuals. Yet few studies have reported results from population‐based samples, leaving open questions about the generalizability of existing findings. Factors proposed to explain transgender individuals’ elevated risk of suicidality derive from several theoretical models (i.e., clinical model, interpersonal model, minority stress model, and societal integration model). These models identify both general risk factors (e.g., mental health risks and interpersonal risks) assumed to be elevated among transgender individuals because of transgender individuals’ exposure to stigma‐related disadvantage and the stigma‐specific risks themselves (e.g., minority stressors such as discrimination). This is one of the first population‐based studies to examine differences in suicidality between transgender and cisgender individuals and theoretically derived factors potentially explaining such differences.

**Methods:**

A sample of 533 transgender and 104,757 cisgender individuals (age 16–84) was analyzed.

**Results:**

Compared to cisgender individuals, transgender individuals were at a substantially higher risk of reporting both lifetime and past 12‐month suicidality. Several factors partially mediated the increased risk of suicidality among transgender compared to cisgender individuals, including depressive symptoms, lack of social support, and exposure to discrimination.

**Conclusions:**

This study suggests that transgender people experience multiple psychosocial health threats and calls for interventions to reduce these threats.

## INTRODUCTION

Results from many studies have suggested that suicidality is more common among transgender people (i.e., individuals who experience incongruity between their sex assigned at birth and current gender identity), compared to the general, presumably cisgender, population (Bränström & Pachankis, 2020; Connolly et al., 2016; Dhejne et al., 2016; Haas et al., 2010; Winter et al., 2016). Yet, despite a recent increase in studies focusing on the mental health of transgender individuals, most have been conducted in small non‐representative samples (Reisner et al., 2016). Therefore, reported rates of suicidal ideation and suicide attempts vary considerably between studies. As an illustration, one systematic review of suicidality among transgender people reported that rates of suicidal ideation ranged from 37% to 83% across studies, whereas rates of suicide attempt ranged from 9.8% to 44% (McNeil et al., 2017). Such wide discrepancies suggest that included studies might be reflecting the experiences of quite different samples of transgender individuals. Population‐based sampling can overcome this limitation while also providing comparisons to the prevalence of suicidality among cisgender individuals and examining psychosocial determinants as predictors of the disparities in suicidality between transgender and cisgender individuals (White Hughto et al., 2015).

Multiple factors have been suggested to explain the higher risk for suicidality among transgender people. First, transgender people's higher exposure to well‐established mental health precursors to suicidality, including depressive symptoms and substance abuse, is believed to at least partially explain the increased risk of suicidality within this group (Dhejne et al., 2016; Keuroghlian et al., 2015; Reisner et al., 2016; White Hughto et al., 2015). According to the *clinical model of suicidality*, psychiatric illness and impulsivity together determine risk for suicidal behavior, in particular among individuals with a lifetime history of aggressive behavior, substance abuse, and childhood experience of abuse (Mann et al., 1999). While psychiatric illness increases risk for suicidal ideation, impulsivity and a predisposition for aggressive behavior increases the likelihood of acting on that ideation. As an externalizing mental health problem, substance abuse can be conceptualized as emerging in part from an underlying tendency toward disinhibition and impulsivity (Krueger et al., 2005; Mann et al., 1999), and thus, according to the clinical model of suicidality can serve as a robust predictor of suicidality, especially in combination with other psychiatric illness such as depression.

Second, according to the *interpersonal theory of suicide* (Van Orden et al., 2010), suicidal ideation is caused by thwarted belongingness and perceived burdensomeness, which are conceptualized as feelings of being socially isolated, lacking social support, and a perception of being a burden to others. This theoretical model has recently been used in one study to explain the increased risk of suicidality among transgender individuals (Testa et al., 2017). However, because that study used a non‐probability sample without a cisgender comparison group, differences in these interpersonal predictors between transgender and cisgender individuals and their role in explaining the disparities in suicidality between these two populations were not possible.

Third, transgender individuals are exposed to stigma‐related stress not experienced by the general population (Hatzenbuehler & Pachankis, 2016; White Hughto et al., 2015). The *minority stress model*, originally developed to explain differences in mental health based on sexual orientation (Meyer, 2003), has in recent years expanded to facilitate understanding of the increased risk of mental health problems based on transgender status as well (Hendricks & Testa, 2012; Operario et al., 2014; Testa et al., 2015; White Hughto et al., 2015). According to the gender minority stress model, stressors related to the stigma associated with belonging to a minority group negatively affects the mental health of transgender individuals and can at least partially explain the elevated risk of suicidality experienced by transgender people (Hatzenbuehler & Pachankis, 2016; Hendricks & Testa, 2012; Testa et al., 2017). One study assessing both internal and external minority stress factors in a non‐probability sample of transgender individuals showed that stigma‐based internal stressors, such as internalized transphobia and anxious anticipation of being exposed to negative events, were particularly strongly related to suicidality in this group (Testa et al., 2017). Also congruent with the minority stress model, external stigma‐based stressors including exposure to stigma‐based discrimination and violence, have also been linked to suicidality among transgender people (Clements‐Nolle et al., 2006; Nuttbrock et al., 2010; Testa et al., 2017).

Fourth, studies in support of the *societal integration model* (Durkheim, 1897) find that sociological risks reflecting one's lack of attachment to society as a whole explain elevations in suicidal ideation and suicide attempts among other minority populations (e.g., sexual minorities; Bränström et al., 2020). In that study, more frequent suicidal ideation and suicide attempts among sexual minority individuals compared with heterosexual individuals were partially explained by barriers to societal integration, including not being married or living with a partner, not living with children, lacking societal trust, and being unemployed. These barriers to societal integration explained the sexual orientation disparity even over‐and‐above the effect of the clinical, interpersonal, and minority stress‐related risk factors reviewed above (Bränström et al., 2020). Whether such barriers might similarly function as predictors of transgender individuals’ increased risk of suicidality remains yet unknown.

This study aimed to investigate the prevalence of suicidality among transgender individuals, and differences in suicidality between transgender and cisgender individuals, using a population‐based probability sample in Sweden. The study also examined several key components of each of the four models of suicidality described above: clinical model (i.e., depression and substance abuse), interpersonal model (i.e., lack of social support), minority stress model (i.e., discrimination and threat of violence), and societal integration model (i.e., not being married, in a registered partnership or living with a partner, not living with children, lack of societal trust, and being unemployed) as potential explanatory factors of any observed disparities in suicidality.

This study possesses several methodological strengths capable of advancing knowledge of the transgender‐cisgender disparity in suicidality. First, this study represents one of the few population‐based examinations of the mental health of transgender individuals, with a comparison group of cisgender participants and a large‐enough sample of transgender individuals to enable an exploration of multiple factors contributing to the increased risk of suicidality within this group. Second, this study assessed a comprehensive set of risk factors for suicidality, thereby permitting a simultaneous test of key components of four theories of suicidality: the clinical model (Mann et al., 1999), interpersonal model (Van Orden et al., 2010), minority stress model (Meyer, 2003), and societal integration model (Durkheim, 1897). Previous studies have only either tested one of these models (Clements‐Nolle et al., 2006; Perez‐Brumer et al., 2015) or two of these models in combination (Testa et al., 2017), but never the integration of models examined here. Further, none have tested any combination of these models using a population‐based sample of transgender individuals.

Taking advantage of these combined methodological strengths, we tested the hypotheses that (1) transgender individuals are at greater risk of suicidality than cisgender individuals, and (2) the increased risk of suicidality among transgender individuals can be partially explained by: (a) more depressive symptoms and substance abuse, (b) lack of social support, (c) greater exposure to discrimination and threat of violence, and (d) barriers to societal integration, drawing upon key predictions of prominent models of suicidality in the general (Durkheim, 1897; Mann et al., 1999; Van Orden et al., 2010) and transgender (Hendricks & Testa, 2012; Testa et al., 2017) population.

## METHODS

### Participants

This study takes advantage of data from the Swedish National Public Health Survey collected in 2018 by the Public Health Agency of Sweden. Invitations to participate in the survey were sent to a random sample of 282,086 people (age 16–84); 117,178 (41.5%) individuals successfully responded. The participants could answer the survey via paper‐and‐pencil or online. The survey collected information about the health and life experiences of the Swedish population and responses were complemented with data about legal gender, age, marital and partnership status, level of education, and income drawn from national registers. Data were weighted to reflect the total population in Sweden in 2018.

### Materials

#### Transgender status

Participants’ transgender status was classified as either transgender or cisgender based on responses to the question “Are you or have you been a trans person?” with a definition: “Trans person is a collective term that usually concerns individuals whose gender identity and/or gender expression sometimes or always deviates from the norm of the gender they were assigned at birth.” The question could be answered with “yes,” “no,” or “I don't know.” Of all participants in this study, 533 (0.5%) responded “yes” and were categorized as transgender, and 104,757 (99.5%) responded “no” and were categorized as cisgender. Individuals who did not answer the question (1.7%) and individuals who answered “I don't know” (0.8%) were not included in further analyses due to unknown transgender status.

#### Suicidality

Four measures of suicidality were used in the current study: lifetime suicidal ideation and suicide attempt and past 12‐month suicidal ideation and suicide attempt. Suicidal ideation was identified using the question: “Have you ever been in a situation where you seriously considered taking your own life?” Suicide attempts were identified using the question: “Have you ever tried taking your own life?” Both questions had the response alternatives “no, never,” “yes, more than 12 months ago” and “yes, during the last 12 months.” From this, four dichotomous variables were created: (1) lifetime suicidal ideation (i.e., having considered suicide more than 12 months ago or during the past 12 months) or not, (2) lifetime suicide attempt (i.e., having attempted suicide more than 12 months ago or during the past 12 months) or not, (3) past 12‐month suicidal ideation or not, and (4) past 12‐month suicide attempt or not.

#### Mental health risks

Mental health risk factors for suicidality included depressive symptoms and substance abuse. Depressive symptoms were measured with a five‐item version of the General Health Questionnaire (GHQ‐5), a frequently used measure of current depression. The GHQ‐5 focuses on two major types of symptoms: the inability to carry out normal functions (e.g., “Over the past few weeks, have you been able to enjoy your normal day‐to‐day activities?” with response alternatives: “more so than usual,” “same as usual,” “less so than usual,” and “much less than usual”) and the presence of distressing experiences (e.g., “Over the past few weeks, have you been feeling unhappy and depressed?” with response alternatives: “not at all,” “no more than usual,” “rather more than usual,” and “much more than usual”). Responses to each item were first coded as indicating the presence of the symptom or absence of the symptom, and the responses to all five items were summed into a total score (range: 0–5). Consistent with prior literature and recommended scoring [23], we created a dichotomous variable categorizing participants into groups with “no elevated depression symptoms (i.e., less than two symptoms)” and “current elevated depression symptoms (i.e., two symptoms or more).” The GHQ has shown adequate validity in general population samples and has demonstrated satisfactory sensitivity and specificity for predicting current major depressive disorder diagnosis [22–24].

Substance abuse was measured as high‐risk alcohol consumption and/or any use of cannabis and/or any use of other narcotics during the past 12 months, creating a dichotomous variable. High‐risk alcohol consumption was measured as drinking at least six units of alcohol during one occasion at least once per month during the past 12 months.

#### Interpersonal risks

Interpersonal risks were operationalized as lack of social support, measured with two questions: “Do you have someone you can share your innermost feelings with and entrust?” with the response alternatives “yes” and “no,” and “Can you get help from any person or people if you have practical problems or are ill?” with the response alternatives “yes, always,” “yes, most of the time,” “no, most of the time not,” and “no, never.” The answers involving “no” were coded as lacking this type of social support. Participants were classified as lacking social support if they had answered “no” to one or both of the social support questions.

#### Minority stress risks

Two items assessed minority stress risks. Exposure to discrimination was measured with the question “In the past 3 months, have you been treated in a way that made you feel discriminated against?” with the response alternatives “no,” “yes, one time,” and “yes, many times,” with the last two answers being categorized as having been exposed to discrimination, creating a dichotomous variable. Exposure to threats of violence was measured with the question “In the past 12 months, have you been subjected to threats of violence so that you were frightened?”, with the response alternatives “yes” and “no.”

#### Societal integration risks

Four variables were used to operationalize barriers to societal integration: (1) not being married, in a registered partnership, or living with a partner, (2) not living with children, (3) lack of societal trust, and (4) being unemployed. Information on marital and partnership status was collected from national registers. Self‐reported household composition was used to categorize participants as living with a partner or not, and as living with children or not. Lack of societal trust was assessed with the question “Do you think one can generally rely on most people?” with the response alternatives “yes” and “no,” with participants answering “no” being categorized as lacking societal trust. Being unemployed was assessed using the question “What is your current occupation?” with “unemployed” being one of the response alternatives.

#### Sociodemographic factors

Age, annual disposable income, level of education (i.e., having a university degree or not), ethnicity (i.e., born in Sweden, born in another European country, or born outside of Europe), urbanicity (i.e., living in a larger city, living in a smaller city, or living in a rural community), and sexual orientation (i.e., heterosexual, bisexual, homosexual, “I don't know,” “other,” and non‐response) were used as covariates in the analyses. Information about legal gender was collected from national registers. Gender identity was assessed using the question: “How do you define your gender identity?” with the response alternatives “woman,” “man,” “other,” and “I don't know.” In a report by the Public Health Agency of Sweden (Public Health Agency of Sweden, 2015), 14% of the respondents included in a targeted non‐probability sample of transgender individuals reported having changed their legal gender, and more than a third of the participants reported that they wanted to change their legal gender. Since we did not have access to information regarding change of legal gender in the current study, this variable was not used as a covariate; some participants may have changed legal gender, while others may not have done so. We did not use gender identity as a covariate given its potential association with transgender status.

### Statistical analyses

Descriptive statistics were used to examine sociodemographic differences between transgender and cisgender participants. Unadjusted and adjusted logistic regression analyses were then used to estimate differences between transgender and cisgender participants in terms of suicidality and the proposed risk factors. Next, we examined whether mental health, interpersonal, minority stress, and societal integration risks explained or partially explained disparities in suicidality between transgender and cisgender participants using multiple mediation analyses. For the multiple mediation analyses, all nine proposed mediating variables drawn from the theoretical models were included: mental health risks (i.e., depression, substance abuse), interpersonal risks (i.e., lack of social support), minority stress risks (i.e., discrimination, victimization/threats), and societal integration risks (i.e., not married/living with a partner, not living with a child, lack of societal trust, and unemployment). To statistically test mediation, we calculated the indirect effects of each variable as a mediator of the association between transgender status and past 12‐month suicidality. Analyses were performed using MPlus using maximum likelihood parameter estimates with robust standard errors (MLR) to calculate direct and indirect effects with 95% confidence intervals. A significant indirect effect (*p* < 0.05) was interpreted as evidence of mediation. To provide information regarding the relative explanatory potential of our variables, we performed stepwise multiple mediation models to calculate the unique proportion of the disparity that was explained by each block of proposed mediators: the mental health risks, interpersonal risks, minority stress risks, and societal integration risks.

There was a total non‐response rate of 0.0%–3.5% across the variables used in this study, with the highest non‐response rates on the questions concerning sexual orientation (3.5%), depressive symptoms (1.7%), and substance abuse (1.8%). Only respondents with complete data on all outcome and predictor variables were included in analyses. All statistical analyses were performed using post‐stratification weights to adjust for selection probabilities and non‐response. Analyses were performed using SPSS version 24 and Mplus Version 8.5.

## RESULTS

### Descriptive statistics

Sociodemographic descriptives of the sample and differences between transgender and cisgender individuals are presented in Table 1. Transgender individuals were younger (range: 16–84; median = 40.0; mean = 43.0 [SD = 18.8], *t* = 6.75, *p* < 0.001) compared with cisgender individuals’ (range: 16–84; median = 48.0; mean = 47.8 [SD = 18.5]). Transgender participants were less likely to have been born in Sweden (*X* ^2^ = 70.28, *p* < 0.001); more likely to be born outside of Europe (*X* ^2^ = 136.36, *p* < 0.001); and more likely to have a female legal gender, report not knowing their gender identity (*X* ^2^ = 321.91, *p* < 0.001), and report another gender identity than woman or man (*X* ^2^ = 8442.82, *p* < 0.001), and therefore also less likely to report man (*X* ^2^ = 32.73, *p* < 0.001) or woman (*X* ^2^ = 6.99, *p* = 0.008) as their gender identity, compared with cisgender participants. Moreover, compared with cisgender participants, transgender participants were more likely to report being bisexual, being homosexual, or being uncertain of their sexual orientation, to report “other” as their sexual orientation, and to not respond to the question about sexual orientation (*all p* < 0.001). Additionally, transgender individuals had a lower annual income and level of education than cisgender individuals. Urbanicity did not differ significantly between groups.

**TABLE 1:** Sample characteristics by transgender status

	Cisgender *n* = 104,757	Transgender *n* = 533	
Age, years	*n* (%^a^)	*n* (%^a^)	*t* = 6.75***
16–25	9330 (14.6)	98 (26.1)	
26–35	11,460 (15.9)	82 (18.5)	
36–45	13,525 (16.8)	49 (9.4)	
46–55	17,008 (15.8)	75 (15.8)	
56–65	19,159 (16.5)	94 (17.6)	
66–75	23,498 (13.7)	88 (7.6)	
76–84	10,777 (6.6)	47 (4.9)	
Individual income	mean (SD^b^)	mean (SD^b^)	
Mean annual income in SEK	257,998 (387 735)	200,094 (262 501)	*t* = 3.86***
Education	*n* (%^a^)	*n* (%^a^)	*X* ^2^ = 17.03***
University degree	28,477 (25.1)	112 (18.2)	
Country of birth	*n* (%^a^)	*n* (%^a^)	*X* ^2^ = 136.59***
Sweden	92,860 (81.7)	403 (69.2)	
Other European country	7229 (9.3)	52 (8.9)	
Non‐European country	4668 (9.0)	78 (21.9)	
Legal gender	*n* (%^a^)	*n* (%^a^)	*X* ^2^ = 5.02*
Woman	56,839 (49.6)	291 (53.9)	
Man	47,918 (50.4)	242 (46.1)	
Gender identity	*n* (%^a^)	*n* (%^a^)	*X* ^2^ = 8764.33***
Woman	56,613 (49.6)	227 (44.4)	
Man	47,523 (50.2)	223 (39.0)	
Other	55 (0.1)	64 (13.8)	
Do not know	104 (0.1)	17 (2.8)	
Sexual orientation	*n* (%^a^)	*n* (%^a^)	*X* ^2^ = 1369.05***
Heterosexual	97,273 (91.6)	335 (57.6)	
Bisexual	1898 (2.5)	55 (12.7)	
Homosexual	802 (1.0)	30 (9.3)	
Uncertain	1972 (2.2)	33 (6.3)	
Other	500 (0.6)	43 (7.5)	
No answer	2312 (2.1)	37 (6.7)	
Urbanicity	*n* (%^a^)	*n* (%^a^)	*X* ^2^ = 3.17
Larger city	34,083 (34.5)	195 (37.0)	
Smaller city	26,966 (33.8)	128 (30.7)	
Rural community	43,708 (31.7)	210 (32.3)	

*Significant at *p* < 0.05; **significant at *p* < 0.01; ***significant at *p* < 0.001.

^a^ Weighted percentages.

^b^ Standard deviation.

### Differences in suicidality and risk factors between transgender and cisgender participants

Transgender individuals were significantly more likely to report both lifetime suicidality (suicidal ideation: 36.1%; suicide attempt: 15.5%) and 12‐month suicidality (suicidal ideation: 13.3%; suicide attempt: 1.9%) compared with cisgender individuals (lifetime suicide ideation: 12.5%; lifetime suicide attempt: 3.6%; 12‐month suicidal ideation: 3.2%; 12‐month suicide attempt: 0.4%; Table 2). Mental health risks (i.e., depressive symptoms), interpersonal risks (i.e., lack of social support), minority stress risks (i.e., exposure to discrimination and threat of violence), and societal integration risks (i.e., not being married/partnered and not living with children) were more common among transgender individuals than cisgender individuals. However, no significant difference between the groups was found for lack of societal trust and unemployment in adjusted analyses. Further, in adjusted analyses, transgender individuals were less likely to report substance abuse compared with cisgender individuals.

**TABLE 2:** Logistic regressions with associations between transgender status and suicidal ideation and suicide attempts and clinical, interpersonal, minority stress, and societal integration factors

	Cisgender	Transgender	Logistic regression results		
			Cisgender	Transgender	
	*n* (%)^a^	*n* (%)^a^	Reference	OR (CI 95%)	AOR^b^ (CI 95%)
Lifetime suicidal ideation	10,934 (12.5)	135 (36.1)	1	3.97*** (3.38, 4.65)	3.66*** (2.59, 5.17)
Lifetime suicide attempt	3183 (3.6)	60 (15.5)	1	4.86*** (3.93, 6.00)	4.04*** (2.37, 6.88)
Suicidal ideation past 12 months	2522 (3.2)	56 (13.3)	1	4.68*** (3.73, 5.86)	3.86*** (2.47, 6.04)
Suicide attempt past 12 months	267 (0.4)	10 (1.9)	1	5.39*** (3.07, 9.47)	3.45**(1.40, 8.50)
Depressive symptoms	14,506 (16.2)	137 (30.6)	1	2.277*** (1.93, 2.69)	1.99*** (1.40, 2.82)
Substance abuse past 12 months	14,249 (16.3)	66 (11.5)	1	0.67** (0.53, 0.85)	0.65* (0.43, 0.98)
Lack of social support	12,455 (13.2)	100 (24.0)	1	2.08*** (1.74, 2.48)	1.77** (1.17, 2.68)
Exposure to discrimination	20,145 (22.2)	151 (37.5)	1	2.10*** (1.80, 2.46)	1.89*** (1.37, 2.61)
Exposure to victimization or threat of violence	4235 (5.2)	60 (10.0)	1	2.01*** (1.56, 2.59)	1.72* (1.06, 2.80)
Not married, in partnership or living with a partner	30,500 (35.3)	217 (48.8)	1	1.74*** (1.50, 2.03)	1.52* (1.04, 2.23)
Not living with children	61,641 (59.9)	367 (75.9)	1	2.10*** (1.76, 2.51)	2.04*** (1.48, 2.80)
Lack of societal trust	22,698 (26.2)	150 (27.6)	1	1.07 (0.90, 1.27)	0.82 (0.58, 1.18)
Unemployed	2439 (3.6)	29 (6.1)	1	1.76*** (1.28, 2.42)	1.21 (0.70, 2.11)

*Significant at *p* < 0.05; **significant at *p* < 0.01; ***significant at *p* < 0.001.

^a^ Weighted percentages.

^b^ Odds ratios adjusted for age, level of education, country of birth, annual income, and urbanicity.

### Psychosocial determinants as mediators of the difference in suicidality between transgender and cisgender people

**FIGURE 1:**
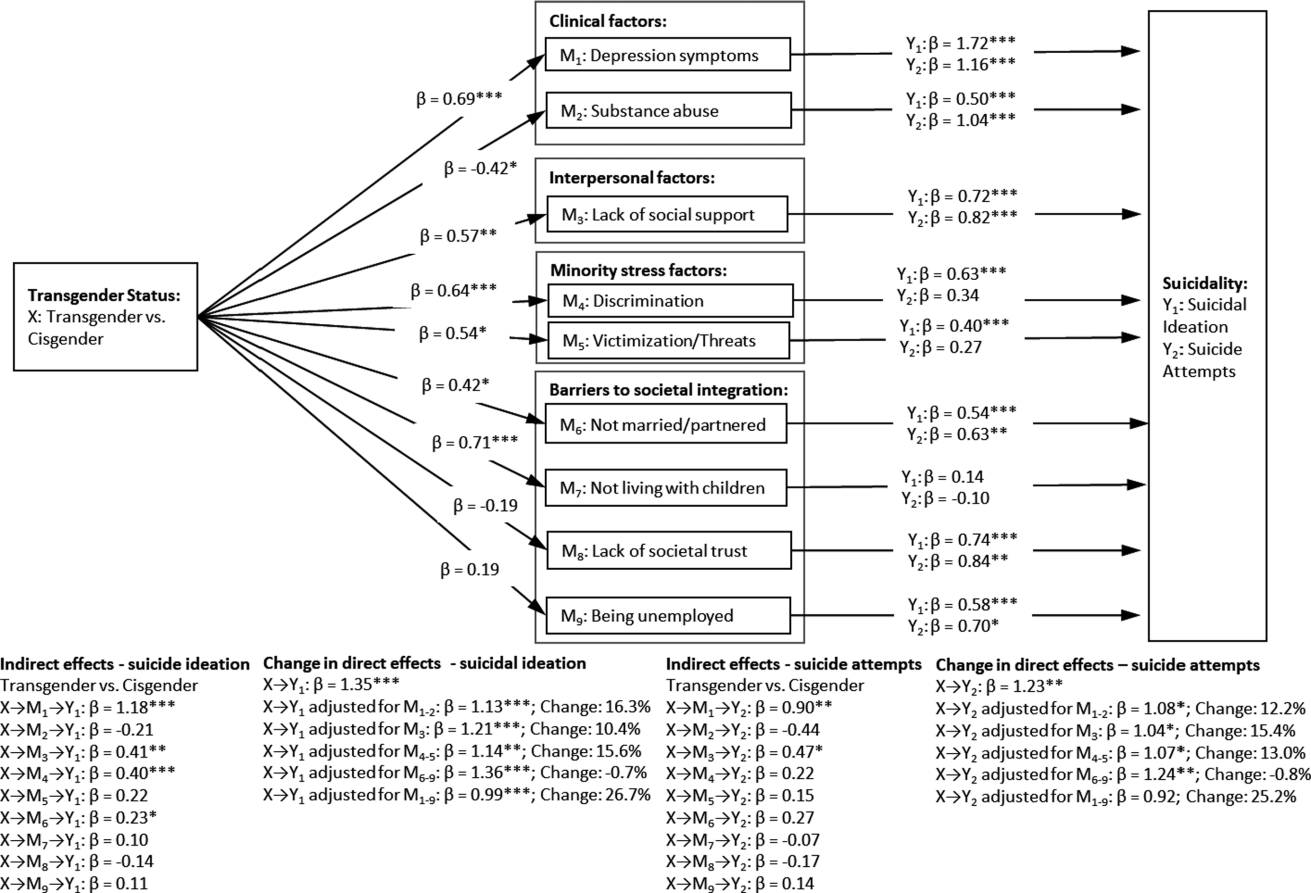
Indirect and direct effect of differences in past 12‐month suicidality between transgender and cisgender participants mediated through clinical, interpersonal, minority stress, and societal integration variables

Results from the multiple mediation analysis showed that most of the examined risks including mental health, interpersonal, minority stress, and societal integration, were significantly associated with past 12‐month suicidality (Figure 1). The only non‐significant associations were between suicidality (both suicide ideation and attempts) and not living with children, and between 12‐month suicide attempt and discrimination and violence. Depressive symptoms and lack of social support showed the strongest indirect effect of the association between transgender status and both past 12‐month suicidal ideation and suicide attempts. For both outcomes, the direct effect of transgender status on suicidality was reduced by more than 25% in models adjusted for all mediators, suggesting that the mediators derived from our four different explanatory model explain just over one‐fourth of the increased risk of suicidality among transgender participants.

To explore the relative importance of the risk factors based on our four proposed theoretical models of suicidality, we calculated the change in direct effect between transgender status and suicidality when our different groups of mediators (i.e., clinical, interpersonal, minority stress, and societal integration) were added. We report the explained variance for each group of mediators (see Figure 1). For suicidal ideation, the largest explained variance was found when the clinical mental health factors (16.3%) and the interpersonal factor (15.6%) were entered as mediators. For suicide attempts, the largest explained variance was found when the interpersonal factor (15.4%) and the minority stress factors (13.0%) were entered as mediators. Our results indicate that societal integration does not contribute to the explanation of the difference in suicidality based on transgender status.

## DISCUSSION

This study provided the unique opportunity to investigate the prevalence of suicidality among transgender individuals compared with cisgender individuals using a population‐representative sample and to explore the relevance of theoretically derived risks for suicidality as explanations of the suicidality disparity between these two groups. Our results showed that 35% and 13% transgender individuals reported lifetime and past 12‐month suicidal ideation, respectively, and 16% and 2% reported lifetime and past 12‐month suicide attempts, respectively. As hypothesized, and in line with prior research using non‐probability samples (Connolly et al., 2016; Public Health Agency of Sweden, 2015; Winter et al., 2016), transgender individuals were at a substantially greater risk of having experienced both suicidal ideations and attempted suicide compared with cisgender individuals. Further, transgender people reported a higher prevalence of most of the risk factors of suicidality examined here, including those derived from theoretical models of clinical risk (i.e., depression), interpersonal risk (i.e., lack of social support), minority stress risk (i.e., discrimination, victimization/threats), and societal integration risk (i.e., not being partnered, and not living with children). These risks, together, partially explained the increased risk of suicidality among transgender individuals compared with cisgender individuals. Although the increased risk of suicidality among transgender people and some of its explanatory factors have been proposed and partially been supported by previous smaller non‐probability studies (Public Health Agency of Sweden, 2015; Reisner et al., 2016; White Hughto et al., 2015), this is, to our knowledge, the first study to report the increased population prevalence of suicidality among transgender people as compared with cisgender people and to demonstrate the explanatory value of a comprehensive set of theoretically derived risks in explaining this disparity.

Since few population‐based studies exist of suicidality among transgender people, it is difficult to compare prevalence rates from our study with previous findings. Also, prevalence rates for suicidality among transgender people tend to vary greatly between studies, for instance, a systematic review of mostly non‐probability studies showed that between 37% and 78% of transgender individuals report a history of suicidal ideation across different studies, and between 9.8% and 44% report a history of suicide attempts (McNeil et al., 2017). Although the prevalence of lifetime suicidal ideation and suicide attempts found in this study falls within the range of these previous studies (i.e., 35% reported suicidal ideation and 16% reported suicide attempts), the prevalence of suicidality in this population‐based sample was closer to the lower end of what is generally reported in studies using non‐probability samples of transgender participants. This is consistent with reviews of the literature on sexual minorities’ risk of suicidality, which find that non‐probability community samples overrepresent individuals at risk for suicide‐related outcomes (Salway et al., 2019).

Findings from this study lend support to three of the four theories of suicide that served as the basis for our exploration as applied to understanding the increased risk of suicidality among transgender people. For instance, the importance of elevated mental health concerns as a risk factor for suicidality, as suggested by the clinical model (Mann et al., 1999), is clearly supported by our findings showing a strong link between depression and both suicide ideation and attempts. The elevated prevalence of depression among transgender people also seemed to explain about 12%–16% of the increased risk of suicidality in this group as compared to cisgender people. In support of the interpersonal model, our results showed that lack of social support, an important aspect of the construct *thwarted belongingness* (Van Orden et al., 2010), was strongly related to suicidality and also significantly mediated the increased risk among transgender people. The addition of social support in our mediation models explained 10.4% of the difference in suicidal ideation and 15.4% of suicide attempts based on transgender status. Further, our results support the minority stress model that describes stigma‐based discrimination and victimization as important risk factors contributing to poor health and risk behaviors among transgender people (Testa et al., 2017). The addition of the minority stress risk factors in our mediation models explained 15.6% of the difference in suicidal ideation and 13.0% of the difference in suicide attempts between transgender and cisgender individuals. However, we did not find that the societal integration model (Durkheim, 1897), which has heretofore only been applied to understanding the elevated suicidality risk among sexual minorities (Bränström et al., 2020), seemed to contribute to transgender people's increased risk of suicidality. Future studies are needed to understand why barriers to societal integration explain sexual orientation differences in suicidality but do not contribute to the understanding of suicidality disparities based on transgender status.

While most of the present findings are consistent with those of previous non‐probability studies (Connolly et al., 2016; Moody & Smith, 2013; Reisner et al., 2016; White Hughto et al., 2015) and existing theories of suicidality (Durkheim, 1897; Mann et al., 1999; Van Orden et al., 2010) as applied to transgender individuals, some of the findings were unexpected. For example, contrary to our hypothesis and findings of previous studies, substance abuse, which has been identified as a risk factor for both suicidal ideation and suicide attempt (Clements‐Nolle et al., 2006; Keuroghlian et al., 2015; Reback & Fletcher, 2014; Reisner et al., 2014), was less common among transgender individuals than among cisgender individuals. This was true despite the fact that sexual minority status, which is known to be associated with higher levels of substance use (Bränström et al., 2020), was more common among transgender individuals. This unexpected finding could be due to the fact that most previous research finding elevated levels of alcohol and illicit drug consumption among transgender individuals has been conducted in the United States (Gilbert et al., 2018), whereas the present study took place in Sweden. Sweden contains a well‐established social welfare system, universal health care, and legislation against workplace and school discrimination targeting one's gender minority status, whereas the United States contains more variable stigma and more variable access to resources depending on socioeconomic and geographic context (International Lesbian Gay Bisexual Trans & Intersex Association, 2019; White Hughto et al., 2016). Another possible explanation for this result could be the fact that few other studies have investigated the prevalence of substance use problems in a representative sample of transgender individuals. Further, in contrast to our prediction, the addition of barriers to societal integration did not seem to contribute to explain the increased risk for suicidality among transgender individuals, but rather seemed to suggest that, given their lower degree of social integration, transgender individuals are somewhat more resilient to suicidality compared with cisgender people. While most barriers to societal integration were associated with suicidality, transgender individuals were only more likely to report not being partnered and not living with children, when controlling for other variables. Notably, when adjusting for sociodemographic factors, transgender individuals were not more likely to report lack of societal trust, a societal integration risk factor associated with both suicidal ideation and suicide attempt. So, while our results support Durkheim's (Durkheim, 1897) theory of societal integration—that being tethered to societal structures of meaning and purpose protect against suicidality risk—the theory did not seem to explain the observed disparities in suicidality between the transgender and cisgender population examined here. However, risks such as low income, depressive symptoms, and lack of social support could actually be consequences of facing barriers to societal integration, and examining barriers to societal integration alongside these factors might have diminished associations.

This study identifies several risk factors for suicidality that are more common among transgender than cisgender individuals, highlighting potential multilevel areas of intervention focus. At the individual level, psychotherapies adapted for transgender populations could address the psychological (i.e., depression) risks of suicidality identified here; some such treatments are currently being evaluated (Budge, 2020). Still, this type of individual‐level intervention could be seen as a way to treat the symptoms rather than the underlying cause. The present findings also suggest that interpersonal interventions, for example, those that promote social inclusion and social support among transgender individuals could reduce transgender individuals’ disproportionate risk of suicide. Such interventions might include mentoring, peer support, and family‐focused interventions. Such interventions can be effectively embedded within existing LGBTQ community venues or delivered online (Hatzenbuehler & Pachankis, 2016), especially in places where access to brick‐and‐mortar resources might be limited. Providing cisgender family and friends psychoeducation and information on transgender‐related supports and needs, and the significance of interpersonal acceptance and support, could be other ways to support transgender individuals (Budge, 2020; Reisner et al., 2016).

In line with minority stress theory and extensions thereof for gender minority stress (Hendricks & Testa, 2012; Testa et al., 2017), interventions that reduce discrimination by challenging transphobic cultural norms, enacting protective legislation, and creating policies that reduce discrimination at the societal level, might also be reasonable candidates for reducing suicidality according to the present study. While Sweden has been shown to have lower levels of structural stigma (e.g., discriminatory legislation and negative attitudes in the general population) towards gender minority individuals than many other European countries (Bränström & Pachankis, 2021), the results from this study show that more effort is needed in order to improve the life chances of transgender individuals even in such a relatively accepting environment. Such efforts could include further de‐pathologizing transgender individuals, for instance through the appropriate and affirmative provision of gender‐related assessments and treatments (Budge, 2020), as well as actions to enable less rigid gender norms and strivings toward a more widespread acceptance of diverse gender expressions.

### Limitations and future research

This study has several notable strengths including the use of population‐based probability sampling and comprehensive assessments of key components of prominent theories of suicidality. Nonetheless, the study should be considered in light of several limitations. First, the cross‐sectional design does not make it possible to determine directionality or causality. An age‐period‐cohort design could help better infer directional effects of the risk factors examined here. Such a design could also capture longitudinal changes in individuals’ nationally registered or self‐reported gender status and associated experiences potentially related to suicidality or protections thereagainst. Second, the data is mostly self‐reported, introducing potential response bias. Using more objective measures of suicidality, for example, registry‐based data, (Bränström & Pachankis, 2020), could possibly yield different results. Third, the assessments of study outcomes—suicidal ideation and attempt—were created for this study and, although they used wording similar to other suicidality assessments in national health surveys (Kessler et al., 1999), these exact items have not been validated against other indicators of suicidality (e.g., registry‐based data regarding suicidality). Further, these two outcomes do not capture other indicators of suicide risk, including creation of a suicide plan, which can provide a more comprehensive assessment of risk (Kessler et al., 1999). Fourth, the current study permitted limited information about transgender identity, gender expression, and gender‐affirming health care. The measure of transgender status that we used in this study (i.e., one question about being or having been a transgender person) does not reflect the large heterogeneity within the transgender population. A more comprehensive set of specific questions on the transgender experience, gender identity, and gender expression should be included in future studies using probability samples, to extend this knowledge. Similarly, measures of risk factors for suicidality were limited to those that can be assessed of the general population often using single items, and measures of exposure to discrimination and violence were assessed in a way that did not make it possible to know if these experiences were linked specifically to transgender identity, a requirement for any indicator of minority stress. While the assessment approach of the current study allowed us to compare the prevalence of risks between transgender and cisgender individuals and to assess all risks as explanatory factors in the population disparity in suicidality, future research is needed that uses well‐validated scales and to incorporate additional risk factors including those specific to transgender individuals. For instance, minority stress theory specifies identity concealment, anxious anticipation of stigma‐based rejection, internalized bias, and lack of gender affirmation as risks (Hendricks & Testa, 2012). Additional variables for consideration in future studies not assessed in the present study include factors derived from the interpersonal model of suicide (e.g., markers of acquired capability including previous suicide attempts and habituation; Van Orden et al., 2010), and clinical model (e.g., generalized anxiety disorder and social anxiety, as well as impulsivity and lifetime aggressive behaviors; Mann et al., 1999). Future research is needed to incorporate these additional factors to more comprehensively evaluate and compare the four theories examined here. Finally, Sweden has been shown to have lower levels of structural stigma towards LGBTQ individuals compared with many other countries, and a lower degree of country‐level structural stigma has been shown to be associated with higher life satisfaction among transgender individuals (Bränström & Pachankis, 2021). Therefore, results must be interpreted within the specific context of Sweden.

## CONCLUSIONS

Whereas prior research stems mostly from smaller non‐probability studies and has demonstrated a high, but widely varying, risk of suicidality among transgender people, the present study, using a population‐based sampling design and cisgender comparison, shows that transgender individuals are at an increased risk of experiencing suicidal ideation and suicide attempt. Drawing upon key components of four theoretical models of suicide, the present findings also provide insight into possible explanations for this increased risk, including derived from clinical, interpersonal, minority stress, and societal integration models of suicide. Overall, the present findings add to the growing number of studies showing that transgender people experience multiple threats to health (Reisner et al., 2016) and suggest future interventions that address these health threats across individual, interpersonal, and structural levels.

## CONFLICT OF INTEREST

The authors have no conflict of interest to disclose.

## AUTHOR CONTRIBUTIONS

**Richard Bränström:** Conceptualization (lead); Data curation (lead); Funding acquisition (lead); Investigation (equal); Methodology (lead). **Isabella Stormbom:** Investigation (equal). **Morgan Bergendal:** Investigation (equal). **John Pachankis:** Conceptualization (equal); Investigation (equal); Methodology (equal).

## ETHICAL APPROVAL

This study has been approved by the Swedish Ethics Review Authority (registration number 2019‐06335).
